# Comparative evaluation of domain-specific and general-purpose transformer models for Arabic poet classification

**DOI:** 10.1038/s41598-026-54438-8

**Published:** 2026-07-08

**Authors:** Sarah Alnefaie

**Affiliations:** Department of Computer Science, Faculty of Computing and Information Technology, King Abdulaziz University, Jeddah, Saudi Arabia

**Keywords:** Arabic NLP, Arabic poetry, Domain-specific pretraining, Transformer-based models, Poet classification, Literature, Mathematics and computing

## Abstract

Arabic poet classification presents distinctive challenges stemming from the morphological richness and stylistic diversity inherent in both classical and modern Arabic verse. This study conducts an extensive comparative evaluation of several neural language models to assess their ability to represent poetic expression and capture authorial characteristics. Two carefully curated datasets are utilised: *FrequentPoets*, representing prolific authors with extensive verse collections, and *CrossEraPoets*, encompassing poets from distinct historical periods to examine temporal stylistic variation. A comparative evaluation framework is introduced to contrast domain-specific and general-purpose language models across prolific authorship and cross-era stylistic variation. The domain-adapted AraPoemBERT consistently achieves superior performance, attaining 73.11% accuracy (73.00% F1) on *FrequentPoets* and 77.06% accuracy (77.04% F1) on *CrossEraPoets*, whereas the general-purpose GPT-4o demonstrates considerably lower performance under zero-shot and few-shot evaluation settings. The results highlight the significance of domain-adapted pretraining for morphologically complex languages like Arabic and suggest the potential advantage of transformer-based architectures in modelling stylistic and linguistic nuances unique to Arabic verse. These findings also suggest that temporal diversity may play an important role in model generalisation across different poetic styles. The study contributes to Arabic Natural Language Processing (NLP) and digital humanities by enabling computational authorship attribution, stylistic analysis, and cross-historical exploration of Arabic literary heritage. Overall, the proposed framework provides a robust foundation for future research in Arabic poetry analytics and domain-specific language modelling.

## Introduction

Arabic poetry has long occupied a central place in the cultural and intellectual life of the Arab world. For centuries, it has served not only as a form of artistic expression but also as a repository of collective memory, social values, and historical experience. As noted by Allen^1^ and Cachia^2^, the strong oral tradition surrounding Arabic poetry has embedded poetic expression deeply within communal identity and the broader literary heritage of the region.

Across different historical periods, poetic expression in Arabic literature has evolved through diverse linguistic and aesthetic traditions^3,4^. These stylistic transformations often reflect broader intellectual and social developments, meaning that poetry frequently mirrors the changing cultural landscape of its time. The diversity of poetic styles that emerged across these eras therefore provides a particularly rich setting for computational analysis, especially when investigating how stylistic patterns vary across poets and historical contexts.

Within computational linguistics, poet classification can be viewed as a specific instance of authorship attribution, in which the objective is to determine which poet–among a predefined set of candidates–is most likely to have written a given verse based on stylistic and linguistic evidence^5,6^. More broadly, this task relates to the well-established research area of authorship profiling, which focuses on identifying linguistic fingerprints that characterise individual authors through recurring stylistic patterns in text^5–7^.

Despite growing interest in computational literary analysis, Arabic poetry remains a challenging domain for Natural Language Processing (NLP). Arabic is morphologically rich and structurally complex, and poetic language introduces additional difficulty through figurative expression, stylistic density, and prosodic structure^8–10^. Consequently, approaches based solely on handcrafted rules or shallow statistical representations often struggle to capture the deeper stylistic and contextual signals required for reliable poet attribution.

Recent advances in NLP have increasingly shifted the field toward data-driven models capable of learning textual representations directly from large corpora^11,12^. Earlier studies of Arabic poet attribution commonly relied on traditional machine learning algorithms such as Support Vector Machines (SVMs) and Naïve Bayes (NB), using manually engineered lexical features^13,14^. Although these methods achieved promising results in controlled experiments, their ability to generalise across heterogeneous poetic styles and historical periods remained limited.

Deep neural architectures later improved the modelling of sequential textual patterns. Models based on Long Short-Term Memory (LSTM) and Bidirectional LSTM (BiLSTM) networks learn hierarchical representations directly from data and have demonstrated effectiveness in sequential language modelling tasks^15^. However, because architectures based on Recurrent Neural Networks (RNNs) process tokens sequentially–where each time step depends on the previous hidden state–they may incur higher computational costs when analysing long textual sequences^16^.

Transformer architectures, introduced by Vaswani et al^17^., represent a major advancement in sequence modelling through the use of self-attention mechanisms that enable parallel processing while capturing long-range contextual relationships. Transformer-based models have demonstrated strong capabilities in learning linguistic representations that encode subtle stylistic signals, which can function as stylistic fingerprints for authors. Their success across many NLP applications has encouraged researchers to explore their use in Arabic poetry analysis tasks such as meter detection, rhyme classification, poet identification, and stylistic analysis^10,18^.

In Arabic poetic studies, meter detection refers to the identification of the rhythmic pattern that governs a poetic verse. The traditional system of Arabic prosody, known as al-Arud, organises poems into recognised meters (buhur), each defined by particular patterns of long and short syllables that give the verse its distinctive rhythm^10,19^. Computationally, meter detection therefore involves assigning a poetic verse to its corresponding metrical pattern.

At the same time, Large-scale Pretrained Language Models (LLMs) such as GPT-4o have demonstrated broad multilingual capabilities across a wide range of language understanding tasks^20^. Recent surveys have also highlighted the broader evolution of text classification research, moving from traditional machine learning techniques toward transformer-based architectures and, more recently, LLMs capable of generative reasoning^21^. Despite these advances, the application of such models to specialised literary domains–particularly Arabic poetry–remains relatively underexplored.

However, despite the rapid development of both transformer-based models and LLMs, relatively limited research has systematically examined their effectiveness in capturing poet-specific stylistic signals in Arabic poetry. This limitation highlights the need for systematic comparative evaluations of specialised Arabic models and general-purpose language models within literary NLP contexts.

This observation raises an important research question: Can general-purpose LLMs adequately capture poet-specific stylistic signals in Arabic literary texts, or do domain-adapted transformer models trained on Arabic corpora remain more effective for this task?

Motivated by this gap, the present study conducts a comparative evaluation of domain-specific transformer models and general-purpose LLMs for Arabic poet attribution. Two complementary datasets are employed. The first dataset focuses on highly prolific poets with large poetic corpora, enabling the analysis of stylistic distinctions among frequently represented authors. The second dataset includes poets drawn from different historical periods, allowing the investigation of temporal stylistic variation across Arabic poetic traditions.

To provide a comprehensive comparison, this study evaluates a range of state-of-the-art transformer architectures–including AraBERT^12^, MARBERT, and ARBERT^22^, AraELECTRA^23^, CAMeL-BERT^24^, QARiB^25^, ArabicBERT^26^, and the domain-specific AraPoemBERT^18^–alongside the LLM GPT-4o^20^. In addition, two RNN architectures, namely LSTM and BiLSTM, are implemented as baseline models in order to provide a comparative reference against transformer-based approaches.

### Novelty and contributions

This study makes three main contributions. First, it introduces a cross-era evaluation framework for Arabic poet attribution, enabling systematic analysis of stylistic variation across both different poets and historical periods. Second, it provides a comprehensive empirical benchmark of transformer-based architectures–both domain-specific and general-purpose–using two carefully curated Arabic poetry datasets that capture prolific authorship patterns and temporal stylistic diversity. Third, it presents a direct comparison between specialised Arabic models (e.g., AraPoemBERT) and a multilingual LLM (GPT-4o), providing new insights into the role of domain-adapted pre-training in modelling poetic style and authorial characteristics in Arabic literary texts.

The remainder of the paper is organised as follows. The Literature Review section reviews related work on Arabic poetry analysis and text classification. The Methodology section describes the datasets, preprocessing procedures, and model implementations. The Experiments section presents the experimental results. The Discussion section discusses the main findings and their implications for Arabic NLP research. The Ethics section outlines ethical considerations. Finally, the Conclusion section concludes the paper and suggests directions for future work.

## Literature review

### Authorship analysis and author profiling

Authorship analysis has long been an established research area within computational linguistics and stylometry. At its core, the field investigates how patterns in language use can reveal aspects of an author’s identity. The literature typically distinguishes between three closely related tasks. Authorship attribution focuses on identifying which author, from a predefined set of candidates, produced a given text. Authorship verification addresses a related problem by determining whether two texts were written by the same individual. Authorship profiling, in contrast, aims to infer characteristics of writers–such as demographic or psychological traits–based on recurring linguistic patterns in their writing^5,7^.

Early computational research approached authorship attribution primarily through stylometric analysis. In such studies, researchers extracted linguistic indicators including word frequencies, character *n*-grams, syntactic patterns, and measures of lexical richness. These features were then combined with traditional machine learning algorithms such as SVMs and Bayesian classifiers. These stylometric approaches laid the methodological foundation for computational authorship analysis and continue to serve as important baseline techniques in the field^6^.

The systematic evaluation of authorship attribution methods has also been facilitated by community-driven initiatives, most notably the PAN laboratory at CLEF. These shared tasks introduced benchmark datasets and standardised evaluation frameworks that allow researchers to compare modelling approaches under consistent experimental conditions. As a result, PAN has played a significant role in advancing research on authorship identification systems and establishing widely accepted evaluation practices within the community^27,28^.

In recent years, research in authorship attribution has increasingly shifted toward deep learning methods capable of learning stylistic representations directly from raw text. Transformer architectures have been particularly influential due to their ability to capture long-range contextual dependencies and subtle linguistic signals within textual data^17^. For instance, Fabien et al^29^. demonstrated that fine-tuning the BERT model for authorship attribution can significantly improve classification accuracy compared with traditional stylometric approaches.

Beyond BERT-based models, neural language models have also been explored for capturing author-specific stylistic patterns. Kumarage and Liu^30^ showed that neural language models can effectively model distinctive linguistic signatures associated with individual authors. Similarly, Siino and Tinnirello^31^ employed the XLNet architecture combined with data augmentation techniques to analyse author-related linguistic characteristics in social media data. These studies highlight the growing potential of transformer-based and neural language models for modelling stylistic signals relevant to authorship analysis.

Despite recent advances, a key challenge in authorship classification remains the influence of topical content^32^. When training and testing datasets share similar themes, models may rely on the subject matter of the text rather than the author’s underlying stylistic patterns. As a result, models may appear to achieve high accuracy while actually learning topic-related cues instead of genuine stylistic signals. This issue is particularly relevant in poetry, where poets often write about recurring themes associated with specific literary traditions.

To mitigate this issue, recent research recommends evaluating authorship models under distribution shifts, such as changes in topic, genre, or historical period, in order to better distinguish stylistic patterns that genuinely reflect authorial identity^32^. At the same time, the emergence of LLMs has introduced additional challenges for authorship analysis, including stylistic mimicry, potential data contamination from pretraining corpora, and difficulties in generalising reliably across different textual domains^7^.

These challenges are particularly evident in literary domains such as poetry, where stylistic conventions evolve across historical periods and reflect broader cultural and linguistic developments. Consequently, understanding how computational models capture these evolving stylistic signatures remains an important research direction at the intersection of NLP and digital humanities^5,6^.

### Computational analysis of Arabic poetry

Building on the broader literature on authorship analysis, several studies have explored computational approaches to Arabic poetry. Within this broader framework, poet classification can be viewed as a specialised form of authorship attribution applied to literary texts^5^. While much of the existing stylometric research has focused on prose or social media content, literary genres–particularly poetry–introduce additional complexity due to figurative language, rhythmic structures, and distinctive stylistic conventions. These characteristics make Arabic poetry a particularly compelling domain for exploring computational approaches to stylistic attribution.

Arabic poetry presents several well-documented challenges for NLP systems, including complex metrical structures, rich morphological variation, and substantial stylistic diversity^8,9^. Although Arabic NLP has advanced considerably in recent years, the computational analysis of poetic texts–especially for tasks such as author identification–remains relatively underexplored. The following sections review the evolution of computational approaches applied to Arabic poetry, ranging from rule-based methods to modern deep learning and LLMs.

#### Rule-based and classical machine learning approaches

Early computational research on Arabic poetry primarily focused on metrical analysis using handcrafted rules. Alabbas et al^33^. developed *BASRAH*, a system based on Khashan’s numerical approach that converts poetic lines into *‘Arudi* representations. Their system achieved strong performance when applied to fully vocalised poetic text. Similarly, Abuata and Al-Omari^34^ employed regular expression techniques to identify metrical patterns, reporting an accuracy of approximately 82%.

Subsequent research explored pattern-based enhancements to improve rule-based systems. Berkani et al^35^. combined template matching with curated metrical databases and reported accuracies as high as 99.3%. However, such rule-based systems tend to degrade significantly when applied to non-diacritised text or stylistically diverse poetic forms. Despite their strong performance under controlled conditions, rule-based approaches often lack robustness when dealing with noisy or unvocalised poetic data.

Traditional machine learning methods have also been applied to poet classification. Ahmed et al^13^. employed algorithms such as SVMs, Linear Discriminant Analysis, and Sequential Minimal Optimisation with handcrafted textual features to classify 114 poets, achieving an accuracy of 98.25%. Similarly, Al-Falahi et al^14^. used Decision Trees and NB classifiers to model stylistic characteristics associated with individual poets.

While these approaches achieved promising results on curated datasets, their reliance on manually engineered features limited their ability to generalise across different poetic genres and historical periods. These limitations motivated the transition toward deep learning approaches^10,15^.

#### Recurrent neural networks

RNNs have demonstrated strong performance in modelling sequential data such as textual sequences. Mutawa and Alrumaih^36^trained a character-level BiLSTM model for meter classification and reported an accuracy of 97.53% on complete poetic verses, outperforming both LSTM and GRU baselines. Similarly, Shoubaki et al^15^. demonstrated the effectiveness of BiLSTM architectures using a dataset containing approximately one million lines of classical and modern Arabic poetry.

Despite these successes, RNN-based architectures encounter limitations when modelling long-range dependencies due to their inherently sequential processing structure. In such models, each time step depends on the hidden state produced at the previous step, requiring tokens to be processed sequentially rather than simultaneously. This sequential dependency limits parallel computation across sequence elements and may increase computational cost when analysing longer textual sequences. These limitations have motivated researchers to explore transformer-based architectures, which rely on self-attention mechanisms to capture global contextual relationships more efficiently^17^.

#### Transformer-based architectures

Transformer architectures have emerged as leading approaches in Arabic NLP, demonstrating strong performance across a wide range of downstream language tasks^24,25^. Mutawa and Sruthi^10^ evaluated six transformer-based models–including AraBERT, ArabicBERT, CAMeLBERT, MARBERT, ARBERT, and AraELECTRA–for Arabic meter classification. Among these models, CAMeLBERT achieved an accuracy of 90.62 and an F1-score of 0.91.

More recently, Qarah^18^ introduced AraPoemBERT, a domain-specific transformer model pretrained on more than two million lines of Arabic poetry. The model achieved state-of-the-art performance across several poetic analysis tasks, including 99.03% accuracy in meter detection, 97.73% in rhyme classification, and 99.34% in gender classification. Although AraPoemBERT performs exceptionally well on structural poetic tasks, its potential for modelling stylistic and authorial characteristics remains largely unexplored–a gap that the present study seeks to address.

While transformer-based models have shown strong performance in structured poetic tasks, recent advances in LLMs have introduced a new paradigm for language understanding and generation.

#### Large language models

Recent advances in LLMs, particularly models such as GPT-4 and GPT-4o, have significantly improved performance across a wide range of NLP tasks, including text classification and language understanding. For instance, Wang et al^37^. demonstrated that GPT-4 can outperform traditional classifiers in clinical text classification, achieving an accuracy of 94.4%. Although conducted in the medical domain, these findings highlight the strong generalisation capabilities of LLMs across diverse classification settings.

Recent survey studies have also documented the broader evolution of text classification research, which has progressively shifted from traditional machine learning approaches toward transformer-based architectures and, more recently, LLMs capable of generative reasoning^21^. This progression illustrates how modern language models have become increasingly capable of capturing complex linguistic patterns across a wide range of domains.

Within literary contexts–particularly in poetic texts–the ability of LLMs to represent distinctive poetic style remains uncertain. Walsh et al^38^. observed that ChatGPT exhibits limited stylistic variability when generating poetry compared with human authors, raising questions about its capacity to reproduce distinctive authorial styles.

Taken together, the reviewed literature indicates substantial progress in computational approaches to authorship analysis and the automated processing of Arabic poetry. Recent advances in LLMs further suggest promising capabilities for capturing complex linguistic patterns. Nevertheless, important limitations remain in modelling poet-specific stylistic signals, particularly in Arabic poetry and across different historical periods. Addressing these limitations motivates the comparative evaluation conducted in the present study.

### Research gap

Despite the considerable progress achieved in Arabic NLP and computational approaches to literary analysis, several important research gaps remain in the area of Arabic poet classification and stylistic attribution.

- Much of the existing research on Arabic poetry has primarily focused on structural analysis tasks, particularly meter detection and rhyme identification (e.g^33–35^.,). While these studies have significantly advanced the computational analysis of poetic structure, comparatively fewer studies have investigated stylistic attribution or the task of identifying the author of a verse based on linguistic and stylistic features.
- Transformer-based architectures have demonstrated strong performance across a wide range of Arabic NLP applications, including text classification, sentiment analysis, and language understanding. However, their application to Arabic poet classification has received relatively limited systematic investigation, particularly compared with more extensively studied NLP tasks.
- Existing studies rarely provide a direct comparative evaluation between domain-adapted Arabic transformer models and general-purpose LLMs within a unified experimental framework. Consequently, the relative advantages of specialised Arabic language models versus general-purpose LLMs for literary authorship tasks remain insufficiently understood.
- Another aspect that remains insufficiently explored concerns temporal stylistic variation in Arabic poetry. Differences between classical and modern poetic language may influence model behaviour and classification performance, yet the robustness of poet classification systems across historical periods has rarely been examined in depth.

### Contributions

To address these research gaps, this study makes the following contributions:

- First, it introduces a cross-era evaluation framework for Arabic poet classification using two complementary datasets (*FrequentPoets* and *CrossEraPoets*). This framework enables the analysis of stylistic variation across different historical periods of Arabic poetry.
- Second, the study provides a systematic benchmark of several transformer-based models developed for Arabic NLP and compares their performance with RNN baselines, including LSTM and BiLSTM architectures, for the task of Arabic poet classification.
- Third, the study presents a comparative evaluation between domain-adapted Arabic transformer models and a general-purpose LLM (GPT-4o). The evaluation considers both zero-shot and few-shot prompting scenarios in order to examine the impact of domain-specific pretraining on literary text classification.

## Materials and methods

This section describes the datasets, preprocessing procedures, deep learning architectures, and evaluation metrics used for Arabic poet classification. It also outlines the computational infrastructure employed and the training configuration of each model.

### Dataset construction

This study aimed to construct two distinctive datasets to assess model performance in classifying Arabic poetic verses and to explore the impact of temporal variation in poetic language on classification accuracy. The first dataset, **FrequentPoets**, comprises four poets with the highest verse counts, enabling performance evaluation based on prolific output. The second dataset, **CrossEraPoets**, includes four poets from distinct historical periods, designed to assess how temporal linguistic diversity affects model discrimination.

Poetic verses were primarily sourced from the AraPoems dataset^18^, which served as the main reference corpus due to its wide coverage and well-structured format. To ensure data validity and authenticity, the collected content was cross-verified with publicly available Arabic poetry platforms, including *Aldiwan*^39^ and the *Abu Dhabi Poetry Encyclopedia*^40^, through automated web access and manual inspection.

To reduce potential topic bias, the dataset includes poems from different themes and historical periods. This allows the evaluation to focus on stylistic patterns rather than topical similarity.

**Table 1:** Statistics of the FrequentPoets and CrossEraPoets Datasets.

**Dataset**	**Poet**	**Verses Count**	**Historical Era**
*FrequentPoets*	Ibn Al-Rumi	32,066	Abbasid
	Khalil Mutran	23,748	Modern
	Ahmed Al-Safi Al-Najafi	22,703	Modern
	Mihyar Al-Daylami	22,645	Abbasid
*CrossEraPoets*	Ibn Al-Rumi	32,066	Abbasid
	Al-Ma’arri	13,980	Fatimid
	Khalil Mutran	23,748	Modern
	Al-Nabulsi	15,827	Ottoman

### Preprocessing

The preprocessing pipeline included several cleaning and normalisation steps. Duplicate and non-poetic lines were removed, and orthographic variants (e.g., different forms of the letter Alef) were standardised. Stopwords and diacritics were deliberately retained because they play an important role in preserving the rhythmic and metrical structure of Arabic poetry. Removing them could distort stylistic patterns that are essential for capturing poetic expression. In addition, recent studies have shown that extensive preprocessing is not always beneficial for transformer-based architectures, as these models are capable of learning contextual representations directly from raw text^41^. Therefore, only minimal preprocessing was applied to preserve the linguistic and stylistic characteristics of the verses while avoiding unnecessary information loss. Text cleaning was implemented using custom Python scripts and regular expressions to ensure consistency across both datasets.

To ensure a balanced and leakage-free evaluation, the datasets were partitioned in a stratified manner, maintaining the proportion of verses per poet across the training, validation, and testing splits (80%, 10%, and 10%, respectively). Duplicate or overlapping verses shared between *FrequentPoets* and *CrossEraPoets* were eliminated to prevent data contamination. Additionally, poem-level splitting (*poem-aware*) was applied whenever feasible, ensuring that verses from the same poem did not appear in both the training and evaluation subsets. This strategy effectively reduced memorisation bias and ensured a more reliable assessment of generalisation across poets.

Table 1 summarises the statistics of both datasets. In the *FrequentPoets* dataset, the verse counts per poet were as follows: Ibn Al-Rumi (32,066), Khalil Mutran (23,748), Ahmed Al-Safi Al-Najafi (22,703), and Mihyar Al-Daylami (22,645).

The *CrossEraPoets* dataset includes poets from different historical periods: Ibn Al-Rumi (32,066) from the Abbasid period, Al-Ma‘arri (13,980) from the Fatimid period, Khalil Mutran (23,748) from the Modern period, and Abdul-Ghani Al-Nabulsi (15,827) from the Ottoman period, providing a linguistically diverse distribution across eras.

All datasets and source code will be made publicly available on GitHub upon paper acceptance. To illustrate the linguistic and stylistic diversity of the dataset, Table 2 presents several representative verses from different poets included in the corpus, along with their English translations.

**Table 2:**
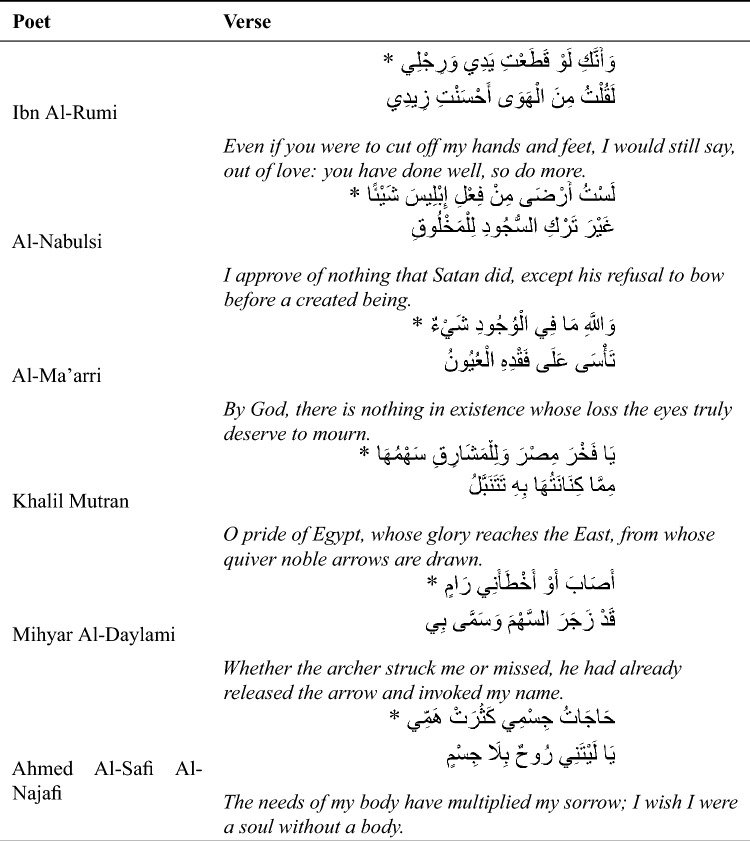
Examples of Arabic poetic verses included in the dataset. Each entry presents a sample verse (as an image) alongside its English translation.

### Classifier workflow

To evaluate poet classification performance, a wide range of deep learning models were examined, encompassing both Transformer-based and recurrent neural architectures. Transformer models included several pre-trained Arabic variants such as ArabicBERT^26^, CAMeL-BERT^24^, QARiB^25^, MARBERT, ARBERT^22^, AraBERT^12^, AraPoemBERT^18^, and AraELECTRA^23^.

In addition, we included a generative LLM (GPT-4o) as well as recurrent neural models (LSTM and BiLSTM)^15^. This diversity enables a comprehensive comparison between transformer-based and traditional sequence models.

Classification was performed separately on the *FrequentPoets* and *CrossEraPoets* datasets. The former concentrates on poets belonging to the same historical era, whereas the latter encompasses distinct time periods to examine how temporal and linguistic variation influences model behaviour.

### Proposed models

#### Pre-trained transformer models

All pre-trained models were fine-tuned on both datasets with a batch size of 32, following best practices established in prior Arabic NLP studies^10^. Each model’s output layer was replaced with a softmax classifier corresponding to the number of poet classes, while maintaining uniform hyperparameters for comparability.

#### GPT-4o prompting setups

We further evaluated the GPT-4o model using two prompting configurations: a zero-shot setup and a few-shot prompt-engineered setup. Both configurations employed Arabic-language prompts instructing the model to identify the poet of a given verse from a predefined list of candidates. The prompts explicitly listed the poet names alongside numerical identifiers. For illustration purposes, the following example is presented in English.

**Zero-shot prompting.** In the zero-shot configuration, the model received only a task instruction together with the list of candidate poets. The model was asked to determine the most likely author of the input verse and return only the corresponding identifier. An example prompt is shown below:

Give me the name of the poet who wrote the following verse, and choose only from this list: ’Ibn Al-Rumi’: 0, ’Khalil Mutran’: 1, ’Ahmed Al-Safi Al-Najafi’: 2, ’Mihyar Al-Daylami’: 3. Verse: “{verse}”. Respond only with the poet’s name from the list above, without any explanation.

**Few-shot prompt engineering.** To further assess whether GPT-4o could benefit from a more explicitly guided inference setting, we conducted an additional experiment based on few-shot prompt engineering. In this configuration, the prompt began with a concise instruction defining the classification task and positioning the model as an expert in Arabic poetry and classical literary styles. The prompt then presented four labelled example verses for each poet before introducing the target verse to be classified. The inclusion of these labelled examples allowed the model to observe representative stylistic and linguistic cues associated with each author, thereby exposing it to recurring poetic patterns that may characterise different poets prior to evaluating the unseen input verse. By combining explicit task-oriented instructions with labelled demonstrations, the prompt provides limited contextual conditioning that may help the model associate salient textual features with the correct poet identity. The purpose of this experiment was to examine whether prompt engineering and few-shot conditioning could partially reduce the performance gap between general-purpose LLMs and specialised domain-adapted transformer models. For conciseness, the full few-shot prompt containing multiple labelled examples is not reproduced here; however, it follows the same overall structure as the zero-shot prompt, with the addition of labelled exemplar verses preceding the target verse.

**Prompt configuration and generation settings.** All GPT-4o experiments were executed using the OpenAI API with *temperature* set to 0.0, default top-p (i.e., 1.0), and a maximum of 20 output tokens. A single response was generated per verse without majority voting. To constrain outputs to the predefined label set, prompts explicitly required the model to return only one poet identifier from the candidate list. All prompts were authored in Modern Standard Arabic; the English example shown here is provided solely for illustration. The prompts were designed following contemporary prompt engineering practices for Arabic NLP tasks^42^.

### Baseline models

Two recurrent baselines were implemented: a unidirectional LSTM and a bidirectional BiLSTM. Each verse was tokenised into sequences of up to 300 tokens, with 128-dimensional embeddings. The LSTM model comprised two stacked layers (128 and 64 units), while the BiLSTM model employed three bidirectional layers to capture contextual dependencies in both directions.

Global Average Pooling was applied to the recurrent outputs, succeeded by a fully connected layer comprising 64 ReLU-activated neurons, and a final softmax classification layer. A dropout rate of 0.3 was used to mitigate overfitting.

In the final classification layer, the softmax activation function is used to convert the model output scores (logits) into a probability distribution over the poet classes^16,43^. The raw logits cannot be interpreted directly as probabilities because they are unbounded scores that may take any real value and do not necessarily sum to one. Therefore, applying softmax is necessary to normalise these scores into comparable class probabilities, which is consistent with the standard cross-entropy loss function commonly used for multi-class classification. In practice, the softmax function is typically combined with the cross-entropy loss, which measures the discrepancy between the predicted probability distribution and the true class label. Since Arabic poet classification is a multi-class classification problem, where each verse belongs to exactly one poet, softmax is particularly appropriate because it assigns mutually exclusive probabilities across all candidate poets and allows the model to select the most likely author.

For an input vector $z = (z_1, z_2, \dots , z_K)$, the probability of class *i* is computed as:

1 $$\begin{aligned} P(y=i)&= \frac{e^{z_i}}{\sum _{j=1}^{K} e^{z_j}} \end{aligned}$$

where *K* denotes the total number of poet classes. This operation ensures that all output probabilities are non-negative and sum to one, making the output directly interpretable as class probabilities. Intuitively, softmax amplifies the differences between logits by assigning higher probabilities to larger values while suppressing smaller ones, enabling clearer class separation.

To illustrate this process, consider a hypothetical example in which the model analyses a poetic verse and produces the following logits for four poets from the FrequentPoets dataset: Ibn Al-Rumi (2.0), Khalil Mutran (1.5), Ahmed Al-Safi Al-Najafi (1.0), and Mihyar Al-Daylami (0.5).

The exponential values are calculated as:

2 $$\begin{aligned} e^{2.0}&= 7.39 \end{aligned}$$

3 $$\begin{aligned} e^{1.5}&= 4.48 \end{aligned}$$

4 $$\begin{aligned} e^{1.0}&= 2.72 \end{aligned}$$

5 $$\begin{aligned} e^{0.5}&= 1.65 \end{aligned}$$

and the total sum becomes:

6 $$\begin{aligned} 7.39 + 4.48 + 2.72 + 1.65 = 16.24 \end{aligned}$$

Thus, the final probabilities are:

7 $$\begin{aligned} P(\text {Ibn Al-Rumi})&= \frac{7.39}{16.24} = 0.46 \end{aligned}$$

8 $$\begin{aligned} P(\text {Khalil\ Mutran})&= \frac{4.48}{16.24} = 0.28 \end{aligned}$$

9 $$\begin{aligned} P(\text {Ahmed\ Al-Safi\ Al-Najafi})&= \frac{2.72}{16.24} = 0.17 \end{aligned}$$

10 $$\begin{aligned} P(\text {Mihyar\ Al-Daylami})&= \frac{1.65}{16.24} = 0.10 \end{aligned}$$

The model therefore predicts the verse as belonging to Ibn Al-Rumi because this class has the highest probability.

Unlike sigmoid activation, which is typically used for binary or multi-label classification, softmax is more suitable in this task because poet classification requires assigning each verse to exactly one poet. Softmax provides a normalised probability distribution across all poet classes and supports single-label decision making, which enables a clear and interpretable decision through the $\arg \max$ selection of the class with the highest posterior probability.

Training was conducted with the Adam optimiser for up to 15 epochs, a batch size of 64, and early stopping based on validation performance.

**Fig. 1:**
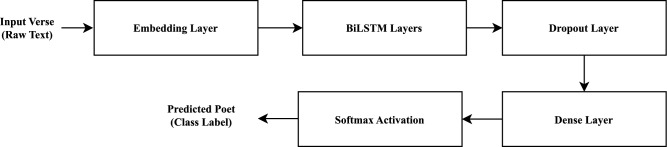
Architecture of the BiLSTM baseline model. The model consists of stacked BiLSTM layers, followed by a global average pooling layer and a dense softmax classifier, enabling it to capture bidirectional contextual dependencies while maintaining computational efficiency.

Figure 1 illustrates the overall BiLSTM structure used as a primary baseline in this study.

### Computational infrastructure

All experiments were executed using the Google Colab cloud environment running Linux with an NVIDIA Tesla T4 GPU and Python 3.8. Initial data preparation (cleaning and organisation) was managed locally under Windows to streamline dataset handling and preprocessing.

#### Optimization and hyperparameters

All transformer-based models, including AraBERT, AraPoemBERT, and related Arabic BERT variants, were fine-tuned under a unified configuration to ensure fair comparison across experiments. The AdamW optimizer was used with an initial learning rate of $2\times 10^{-5}$, a linear decay scheduler with approximately $10\%$ warm-up steps, and a weight decay of 0.01. Training ran for up to four epochs with early stopping triggered when the validation loss failed to improve for two consecutive epochs. A batch size of 32 and a maximum sequence length of 256 tokens were adopted. Tokenisation followed each model’s original vocabulary, with truncation to the maximum length and dynamic padding within each batch. Gradients were clipped at a norm of 1.0, and mixed-precision training (fp16) was applied to enhance GPU efficiency. All experiments used a fixed random seed of 42 to ensure reproducibility, and the checkpoint yielding the best validation Macro-F1 was retained for evaluation.

### Evaluation metrics

To ensure a comprehensive and interpretable evaluation, four standard metrics were adopted: *Accuracy*, *Precision*, *Recall*, and *F1-score*. These metrics are well suited for multi-class classification tasks, where each poet represents a distinct class, and they account for potential class imbalance across poets. Although accuracy reflects overall correctness, it may obscure class-specific disparities; therefore, precision, recall, and F1 were computed per class, with macro-, micro-, and weighted-averaged variants for holistic assessment^10^.

For each poet class *k*, Precision, Recall, and F1-score were computed as follows:

11 $$\begin{aligned} \textit{Precision}_k\;= & \; \frac{TP_k}{TP_k + FP_k} \end{aligned}$$

12 $$\begin{aligned} \textit{Recall}_k\;= & \; \frac{TP_k}{TP_k + FN_k} \end{aligned}$$

13 $$\begin{aligned} \textit{F1}_k\;= & \; \frac{2 \times \textit{Precision}_k \times \textit{Recall}_k}{\textit{Precision}_k + \textit{Recall}_k} \end{aligned}$$

Overall accuracy was computed as:

14 $$\begin{aligned} \textit{Accuracy}&= \frac{\sum _{k} TP_k}{N} \end{aligned}$$

This metric suite provides a balanced and reliable evaluation framework, further supported by confusion matrices and per-class classification reports to visualise model performance. *All reported F1-scores in Tables* 3–6* correspond to the Macro-F1 variant unless otherwise specified.*

## Results

This section presents the experimental results obtained from evaluating multiple models on the *FrequentPoets* and *CrossEraPoets* datasets. It provides a comparative analysis of model performance, highlighting key trends across model architectures, the behaviour of GPT-4o under different prompting strategies, and poet-level classification patterns.

### Performance on the FrequentPoets dataset

Table 3 presents a comparative overview of model performance on the *FrequentPoets* dataset. The results reveal a pronounced disparity between transformer-based models and traditional recurrent neural networks.

The LSTM model achieved a very low F1-score of 10.00%, indicating a limited ability to capture stylistic distinctions within the dataset. The BiLSTM model performed considerably better (F1 = 58.22%), yet it still remained substantially below the performance of transformer-based architectures. These results suggest that sequential architectures may face challenges in capturing the complex stylistic and semantic patterns characteristic of Arabic poetry.

By contrast, transformer-based models–most notably AraPoemBERT–achieved markedly stronger results, underscoring their effectiveness in capturing complex contextual and stylistic signals within poetic language.

AraPoemBERT produced the most competitive outcomes, recording an F1-score of 73.00% and a testing accuracy of 73.11%. In addition to its strong predictive performance, the model also demonstrated computational efficiency, requiring only 19,643.16 seconds for training and 662.03 seconds for inference. Detailed class-level performance metrics are presented in Table 4.

**Fig. 2:**
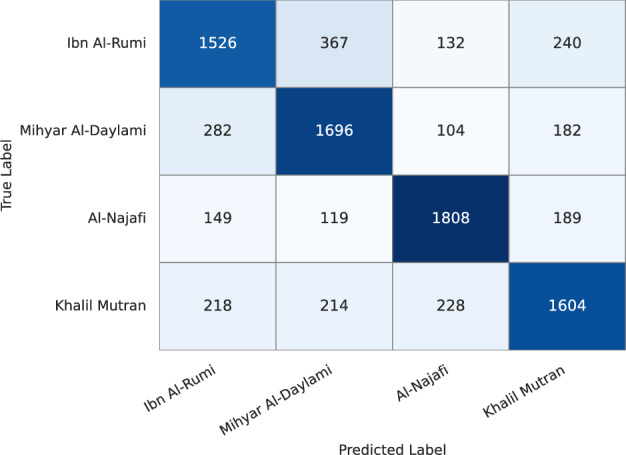
Confusion matrix of AraPoemBERT on the FrequentPoets dataset. Darker cells indicate higher classification counts. The model exhibits strong discriminative performance for Ahmad al-Safi al-Najafi (F1-score = 0.80), whereas verses by Ibn al-Rumi are frequently confused with those of Mihyar al-Daylami and Khalil Mutran, suggesting stylistic overlap.

Among the poets, Ahmad al-Safi al-Najafi was most accurately classified, achieving an F1-score of 0.80, suggesting that his poetic style contains distinctive linguistic patterns that are readily identifiable by the model. In contrast, Ibn al-Rumi obtained the lowest F1-score (0.69), indicating recurring misclassification, particularly with verses attributed to Mihyar al-Daylami and Khalil Mutran. This pattern of confusion is further illustrated in the confusion matrix shown in Figure 2.

Given the relatively low zero-shot performance observed for GPT-4o, an additional experiment was conducted using a few-shot prompting configuration in order to examine whether providing labelled example verses could improve the model’s predictions. In this setup, GPT-4o received prompts that included a small number of labelled example verses for each poet before classifying the target verse.

As reported in Table 3, the few-shot configuration resulted in a modest improvement, with the F1-score increasing from 38.02% to 39.01%. Although the improvement remains limited, it suggests that providing a small number of labelled examples may help the model capture certain stylistic cues associated with different poets. Nevertheless, the results also indicate that such limited contextual guidance may not be sufficient when poets share similar stylistic conventions.

**Table 3:** Comparative results of various models on the FrequentPoets dataset. The table reports training time, evaluation performance, and test metrics for each model. AraPoemBERT achieves the best overall performance across most evaluation metrics.

**Model**	**Train Time**	**Eval Acc**	**Eval Time**	**Test Acc**	**Precision**	**Recall**	**F1**	**Test Time**
LSTM	198.52	25.01	5.75	24.99	6.25	24.99	10.00	1.49
BiLSTM	1666.29	57.65	17.05	58.17	58.46	58.17	58.22	10.31
CAMeL-BERT	59600.41	70.38	2275.68	70.60	70.47	70.60	70.50	2181.94
AraELECTRA	59608.26	68.93	1872.04	69.24	69.06	69.24	68.95	1972.17
MARBERT	62498.97	68.69	1763.75	69.49	69.32	69.49	69.34	4516.35
ARBERT	62188.11	69.03	1842.64	68.39	68.29	68.39	68.31	1855.62
AraBERT	67844.18	63.84	2121.96	63.87	63.61	63.87	63.59	2171.41
QARiB	80607.40	70.87	2777.52	70.55	70.38	70.55	70.36	2816.15
ArabicBERT	55501.96	67.63	1825.51	66.90	66.76	66.90	66.79	1891.88
**AraPoemBERT**	**19643.16**	**73.24**	**627.73**	**73.11**	**73.11**	**73.11**	**73.00**	**662.03**
GPT-4o (Zero-shot)	—	—	—	34.49	48.63	34.49	38.02	25268.22
GPT-4o (Few-shot)	—	—	—	43.22	54.68	43.22	39.01	22142.64

**Table 4:** Class-wise performance of the AraPoemBERT model on the FrequentPoets dataset. The table reports precision, recall, and F1-score for each poet class, demonstrating consistent performance across all categories.

**Poet**	**Poet ID**	**Precision**	**Recall**	**F1-score**
Ibn al-Rumi	0	0.70	0.67	0.69
Mihyar al-Daylami	1	0.71	0.75	0.73
Ahmad al-Safi al-Najafi	2	0.80	0.80	0.80
Khalil Mutran	3	0.72	0.71	0.72

### Performance on the CrossEraPoets dataset

A subsequent experimental phase utilised the CrossEraPoets dataset, encompassing poets representing diverse historical periods. This setup aimed to examine how temporal linguistic variation may influence model discrimination. A comprehensive comparison of model outcomes is presented in Table 5.

AraPoemBERT once again achieved the highest performance, attaining a test F1-score of 77.04% and an accuracy of 77.06%. In addition to its predictive strength, the model exhibited remarkable computational efficiency, requiring only 12,343.09 seconds for training and 426.42 seconds for inference–the lowest among all evaluated transformer-based models.

Among the general-purpose transformer models, QARiB and CAMeLBERT achieved the highest F1-scores (around 75%), while MARBERT followed closely with 72.58%, surpassing AraELECTRA (70.24%) and the remaining baseline models.

Detailed class-level results for AraPoemBERT are presented in Table 6. Among the poets, Abd al-Ghani al-Nabulsi achieved the highest F1-score (0.85), indicating that his verses are more consistently distinguished by the model. By contrast, Abu al-Ala obtained a comparatively lower F1-score (0.74), suggesting greater classification difficulty and a higher degree of stylistic overlap with other poets.

**Fig. 3:**
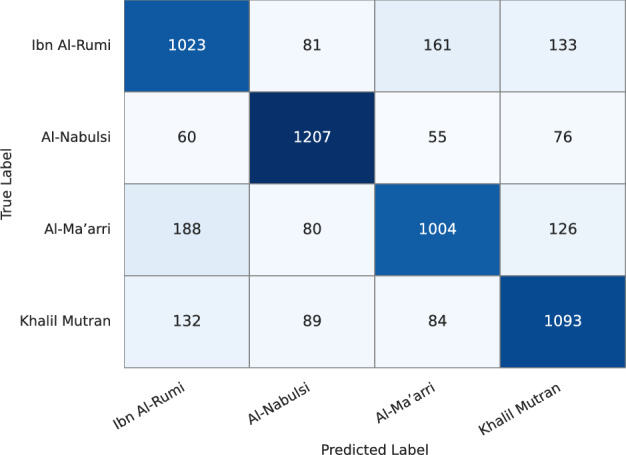
Confusion matrix of AraPoemBERT on the CrossEraPoets dataset. The model accurately distinguishes Abd al-Ghani al-Nabulsi (F1-score = 0.85), whereas verses by Abu al-Ala are frequently misclassified as those of Ibn al-Rumi or Khalil Mutran, suggesting overlapping rhetorical patterns across historical periods.

To further analyse the classification behaviour of AraPoemBERT on the CrossEraPoets dataset, the confusion matrix is presented in Fig. 3. The matrix shows that the model distinguishes Abd al-Ghani al-Nabulsi with particularly high reliability, consistent with the class-level results in Table 6. In contrast, verses attributed to Abu al-Ala exhibit higher confusion rates, particularly with Ibn al-Rumi and Khalil Mutran. This behaviour suggests that certain rhetorical or stylistic similarities between these poets may lead to overlapping representations within the model’s learned feature space. Overall, the confusion matrix demonstrates clearer separation between poets compared with the FrequentPoets dataset, supporting the observation that cross-era stylistic diversity improves classification performance.

Although few-shot prompting improved GPT-4o’s performance on this dataset, increasing the F1-score from 17.16% to 42.68%, the model still remained substantially behind domain-adapted transformer models such as AraPoemBERT. This result highlights the importance of domain-specific pretraining for capturing stylistic patterns in Arabic poetry. It also suggests that stylistic cues provided in the prompt examples may help the model better distinguish between poets when their writing styles originate from clearly different historical periods.

**Table 5:** Comparative results of various models on the CrossEraPoets dataset. The table reports training time, evaluation accuracy, and test performance metrics for each model. AraPoemBERT achieves the best overall performance across all evaluation metrics.

**Model**	**Train Time**	**Eval Acc**	**Eval Time**	**Test Acc**	**Precision**	**Recall**	**F1**	**Test Time**
LSTM	147.05	51.26	4.50	50.39	58.25	50.39	48.65	1.32
BiLSTM	1046.17	61.73	15.11	61.36	62.52	61.36	61.35	5.18
CAMeL-BERT	33379.19	75.18	1118.16	74.12	74.24	74.12	74.04	1080.88
AraELECTRA	50057.09	71.82	1725.55	70.37	70.30	70.37	70.24	1764.71
MARBERT	39363.52	73.82	1185.59	72.51	72.74	72.51	72.58	1225.02
ARBERT	61469.28	71.35	1876.35	71.60	71.63	71.60	71.58	1974.66
AraBERT	37095.60	65.08	1138.49	65.02	64.89	65.02	64.89	1152.72
QARiB	36408.30	75.66	1128.81	75.57	75.61	75.57	75.53	1169.06
ArabicBERT	34674.15	71.05	1191.69	70.42	70.45	70.42	70.40	1186.55
**AraPoemBERT**	**12343.09**	**77.38**	**391.47**	**77.06**	**77.08**	**77.06**	**77.04**	**426.42**
GPT-4o (Zero-shot)	—	—	—	17.44	45.15	17.44	17.16	12001.36
GPT-4o (Few-shot)	—	—	—	47.82	46.66	47.82	42.68	11653.54

**Table 6:** Class-wise performance of the AraPoemBERT model on the CrossEraPoets dataset. The table presents precision, recall, and F1-score for each poet class, indicating robust and consistent classification performance across different historical eras.

**Poet**	**Poet ID**	**Precision**	**Recall**	**F1-score**
Ibn al-Rumi	0	0.73	0.73	0.73
Abd al-Ghani al-Nabulsi	1	0.83	0.86	0.85
Abu al-Ala	2	0.77	0.72	0.74
Khalil Mutran	3	0.77	0.78	0.77

### Representation analysis

To gain further insight into how the model captures stylistic differences between poets, we examined the hidden representations generated by AraPoemBERT for the test sets of both datasets. These representations were obtained from the final encoder layer and projected into a two-dimensional space using Principal Component Analysis (PCA). Each point in these projections corresponds to an individual verse and is labelled according to its associated poet.

**Fig. 4:**
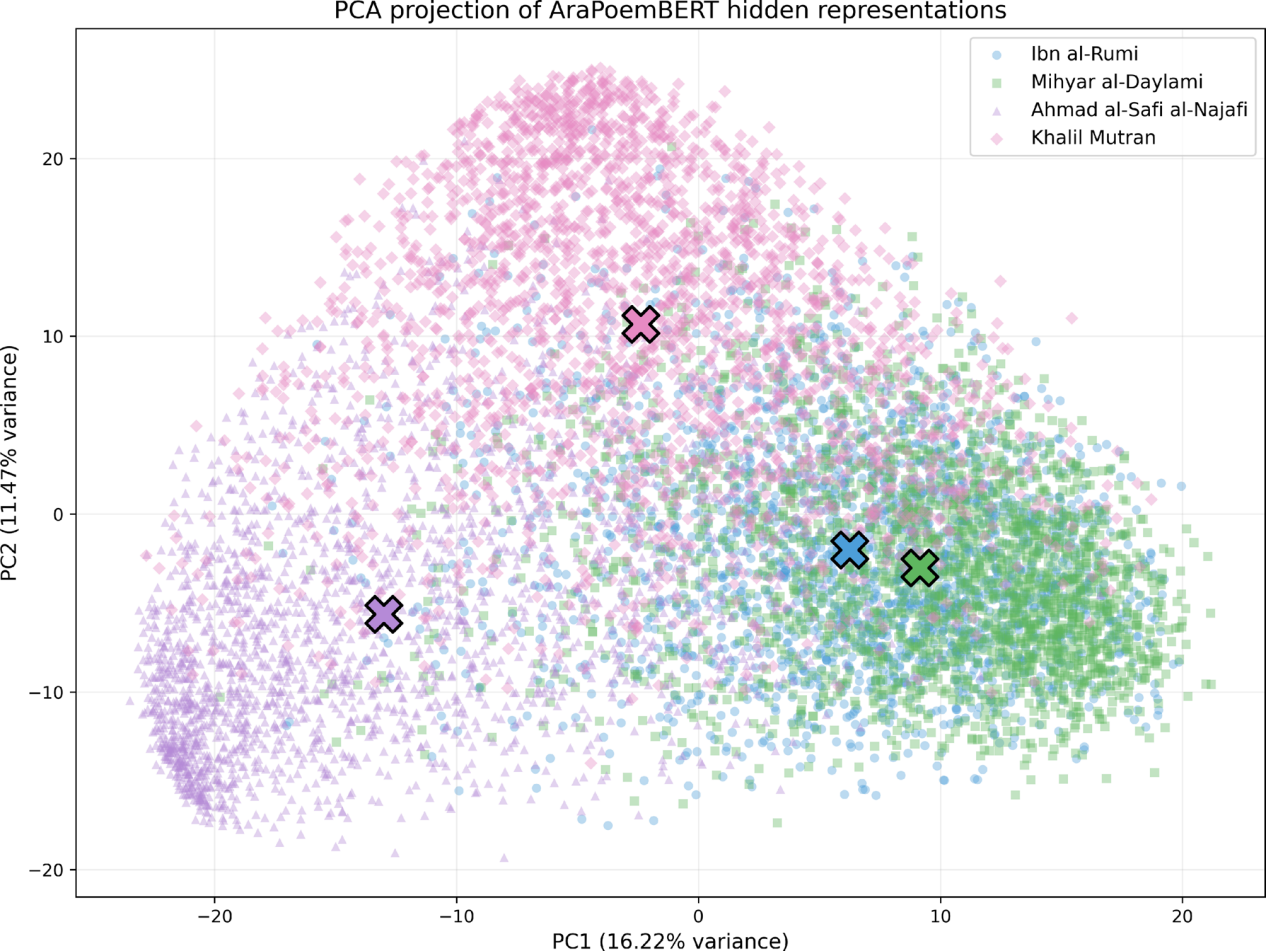
PCA projection of AraPoemBERT hidden representations on the FrequentPoets dataset. Each point represents a verse coloured according to its corresponding poet label. The distribution reveals partially separable stylistic clusters, indicating that the model captures meaningful author-specific patterns in Arabic poetry.

Figure 4 illustrates the PCA projection for the *FrequentPoets* dataset. The resulting distribution reveals partially separable clusters corresponding to different poets. In several regions of the projection space, distinct groupings emerge, indicating that the model captures stylistic patterns associated with specific authors. However, some areas remain partially mixed, reflecting the fact that certain poets share linguistic or rhetorical characteristics, which may contribute to the misclassification patterns observed earlier in the confusion matrices.

**Fig. 5:**
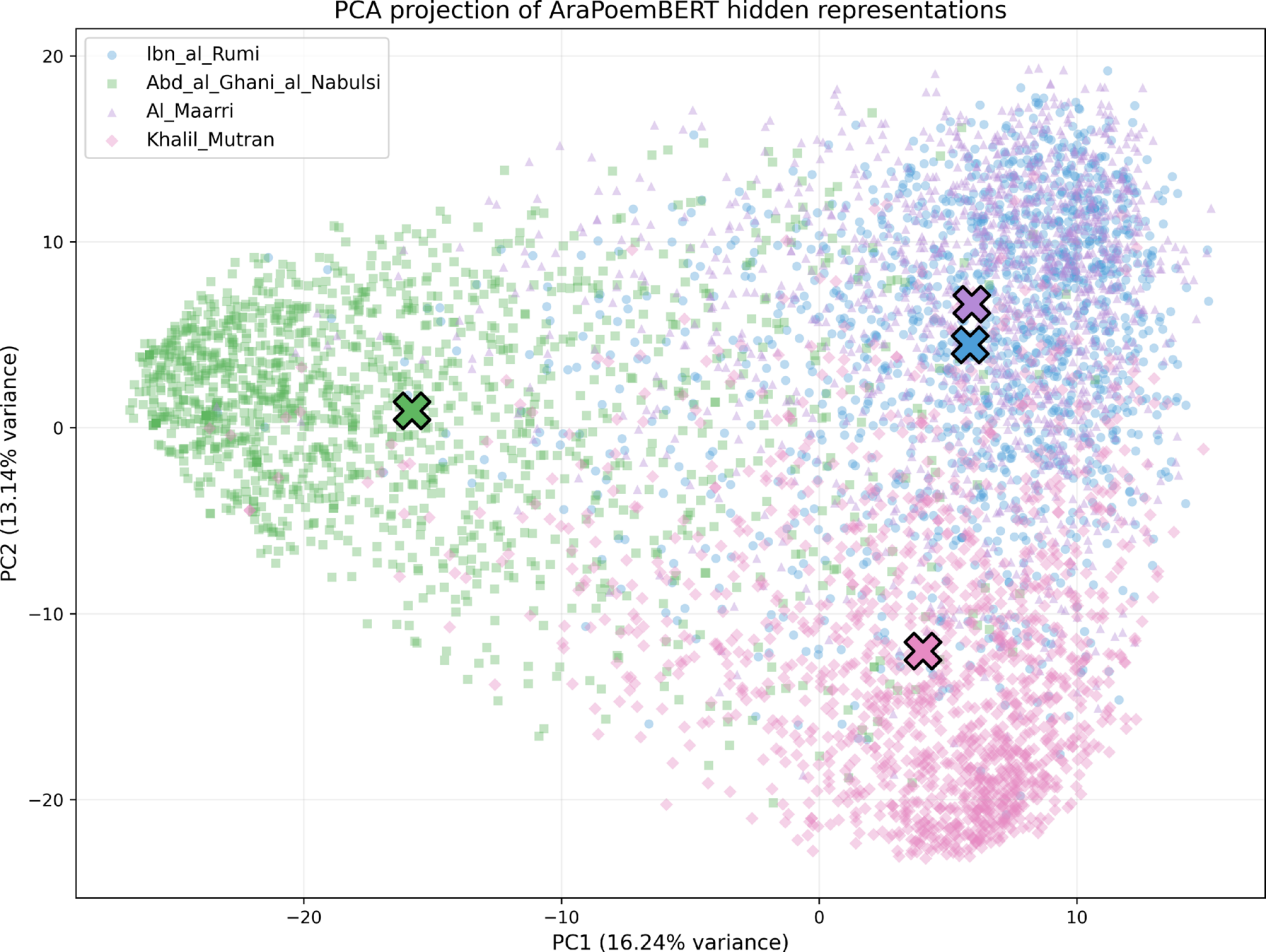
PCA projection of AraPoemBERT hidden representations on the CrossEraPoets dataset. Compared with the FrequentPoets dataset, the clusters appear more clearly separated, reflecting stronger stylistic distinctions between poets from different historical periods.

Figure 5 presents the PCA projection for the *CrossEraPoets* dataset. Compared with the FrequentPoets dataset, the clusters appear more clearly separated, suggesting stronger stylistic distinctions between poets from different historical periods. This observation is consistent with the higher classification accuracy obtained on the CrossEraPoets dataset and indicates that temporal diversity may enhance stylistic separability within the learned representation space.

### Summary of findings

Overall, the experimental evidence highlights AraPoemBERT’s strong performance when compared with both recurrent architectures and other Arabic transformer-based models across the evaluated datasets. Its consistent accuracy and efficiency across poets from different historical backgrounds suggest an effective capacity for capturing stylistic variation in Arabic poetry. Moreover, the model’s rapid inference capability enhances its practicality for real-world literary applications. These findings further indicate the effectiveness of domain-adapted transformer models in capturing the intricate stylistic and linguistic patterns characteristic of classical Arabic poetry.

## Discussion

### Transformer models vs. recurrent baselines

The experimental findings demonstrate a consistent advantage of transformer-based architectures over recurrent neural baselines for Arabic poet classification. While both LSTM and BiLSTM models are capable of modelling sequential dependencies, their performance remains considerably lower than that of transformer-based systems across both datasets (Tables 3 and 5). This result suggests that self-attention mechanisms provide a more effective framework for capturing stylistic signals in Arabic poetry.

Arabic poetic style is rarely expressed through isolated lexical items. Instead, it emerges through complex interactions between distant words, repeated rhetorical constructions, syntactic parallelism, and historically grounded poetic conventions. Transformer architectures are particularly well suited to modelling such characteristics because the self-attention mechanism enables the model to relate distant contextual cues within the same poetic line.

By contrast, recurrent models process sequences incrementally, where each step depends on the previous hidden state. This sequential processing may limit their ability to capture stylistic dependencies distributed across longer spans of text. As a result, transformer models appear better suited to modelling the stylistic structure of Arabic poetry, where authorial signals often emerge through long-range contextual relationships rather than local lexical patterns.

These findings are broadly consistent with prior research in Arabic NLP, where transformer-based architectures have demonstrated strong performance across a range of natural language understanding tasks. Previous studies on Arabic poetry analysis and meter detection have similarly reported promising results when applying transformer models, highlighting their ability to capture contextual and stylistic patterns in poetic language^10,18,24,25^.

### Interpretation of AraPoemBERT performance

Among all evaluated systems, AraPoemBERT achieved the strongest performance across both datasets. On the *FrequentPoets* dataset, the model obtained approximately 73% test accuracy, while performance increased to around 77–78% on the *CrossEraPoets* dataset.

These results indicate that AraPoemBERT consistently outperforms the other evaluated models across both datasets. A plausible explanation for this improvement lies in the domain-adapted pretraining of AraPoemBERT on Arabic poetic corpora. Exposure to large collections of poetic texts during pretraining may allow the model to learn representations that are better aligned with the linguistic and stylistic characteristics of Arabic poetry.

Such domain adaptation can provide the model with a stronger contextual understanding of poetic language, which may support more accurate distinctions between different authors. This advantage is particularly relevant in literary classification tasks, where authorial differences are often subtle and distributed across multiple linguistic signals.

Overall, the results suggest that domain-specific pretraining can play an important role in improving model performance in specialised literary analysis tasks.

### Poet-level misclassification patterns

Further insight into model behaviour can be obtained by analysing the confusion matrices presented in Figs. 2 and 3. In both datasets, the strong diagonal dominance indicates that the model successfully captures author-specific stylistic signals. Nevertheless, several poets exhibit partial confusion with particular counterparts. For example, in the *FrequentPoets* dataset, verses attributed to Ibn al-Rumi are occasionally classified as those of Mihyar al-Daylami or Khalil Mutran, suggesting certain stylistic similarities among these poets. A comparable pattern is observed in the *CrossEraPoets* dataset, where some verses by Abu al-Ala al-Ma’arri are occasionally confused with those of Ibn al-Rumi or Khalil Mutran. Such misclassifications may reflect genuine stylistic proximity between poets rather than purely algorithmic limitations, a phenomenon commonly observed in authorship attribution studies^5^.

To further examine stylistic variation across poets, a lexical diversity analysis was conducted using the type–token ratio (TTR), which measures the proportion of unique words relative to the total number of tokens^44^. The results are summarised in Tables 7 and 8. In the *FrequentPoets* dataset (Table 7), Mihyar al-Daylami exhibits the highest lexical diversity, followed by Ibn al-Rumi and Khalil Mutran, whereas Ahmad al-Safi al-Najafi shows the lowest lexical diversity. Similarly, in the *CrossEraPoets* dataset (Table 8), Khalil Mutran and Abu al-Ala al-Ma’arri display relatively high lexical diversity, while Abd al-Ghani al-Nabulsi demonstrates noticeably lower lexical variation.

Interestingly, when these observations are considered alongside the confusion matrices, a clear tendency becomes observable in the model predictions. Poets with comparatively lower lexical diversity, such as Abd al-Ghani al-Nabulsi and Ahmad al-Safi al-Najafi, tend to exhibit clearer classification behaviour. A possible explanation is that lower lexical diversity may correspond to more consistent lexical or stylistic usage patterns within a poet’s works, which can provide stronger and more stable cues for the model during authorship classification. Previous research in stylometry suggests that consistent stylistic markers can facilitate author identification, as models can more easily capture recurring linguistic patterns associated with a particular writer^45^.

By contrast, poets with higher lexical diversity may employ a broader range of vocabulary and stylistic constructions, potentially leading to more distributed signals within the representation space and slightly higher levels of classification confusion. These findings suggest that lexical diversity may influence the separability of poetic styles in representation space, although it is unlikely to be the sole factor affecting classification performance. Rather, the results indicate that lexical consistency may contribute to clearer stylistic signals that transformer-based models such as AraPoemBERT can exploit during poetic authorship attribution.

**Table 7:** Lexical diversity measured using type–token ratio (TTR) for poets in the FrequentPoets dataset. Higher TTR values indicate greater lexical diversity within each poet’s corpus.

**Poet**	**Corpus TTR**
Mihyar al-Daylami	0.691
Ibn al-Rumi	0.684
Khalil Mutran	0.680
Ahmad al-Safi al-Najafi	0.622

**Table 8:** Lexical diversity measured using type–token ratio (TTR) for poets in the CrossEraPoets dataset. Higher TTR values indicate greater lexical diversity within each poet’s corpus.

**Poet**	**Corpus TTR**
Khalil Mutran	0.731
Abu al-Ala al-Ma’arri	0.729
Ibn al-Rumi	0.726
Abd al-Ghani al-Nabulsi	0.637

Taken together, these observations suggest that lexical diversity alone does not fully explain the model’s classification behaviour. Instead, the results indicate that AraPoemBERT relies on multiple complementary signals when distinguishing between poets, including lexical diversity, recurrent stylistic patterns, token-level importance signals, and contextual representations learned through transformer-based encoding. The interaction of these factors appears to contribute to the separability of poetic styles observed in the confusion matrices and representation-space visualisations.

### Token-level stylistic signals

Beyond lexical diversity, analysing the most influential tokens identified by the model provides deeper insight into how stylistic signals are captured during poet classification. To investigate this aspect, we examined the token-importance scores generated by AraPoemBERT and extracted the most influential tokens associated with each poet across both datasets.

**Fig. 6:**
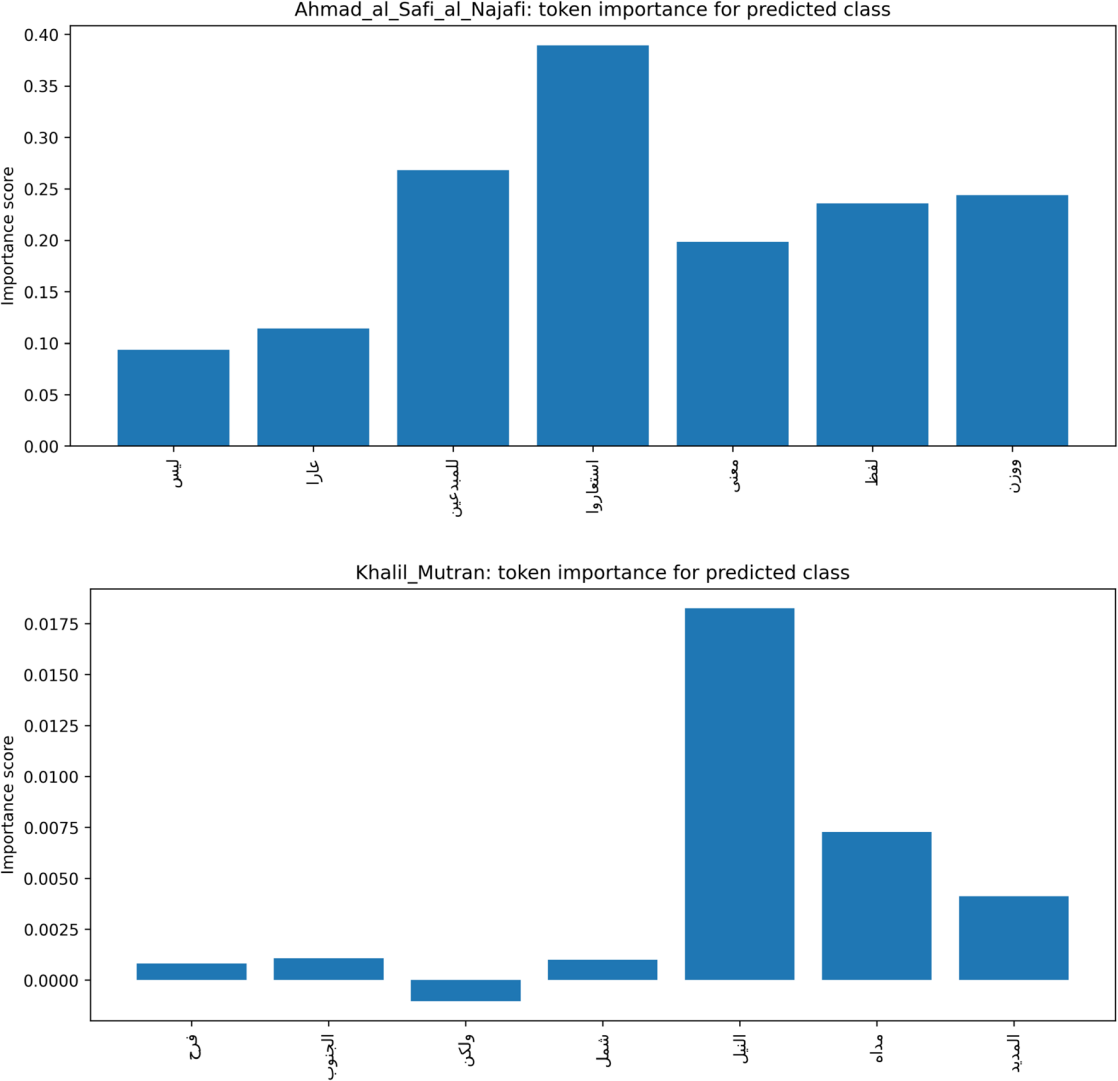
Token importance scores derived from AraPoemBERT for poet classification. (**a**) Ahmad al-Safi al-Najafi, where highly weighted tokens are associated with rhetorical language and poetic terminology such as “isti’ara”, “lafz”, and “wazn”. (**b**) Khalil Mutran, where influential tokens emphasise descriptive imagery and geographical references such as “al-Nil” and “al-janub”. The importance values indicate the contribution of each token to the model’s prediction.

Figure 6 presents representative examples of influential tokens identified by AraPoemBERT for two poets, illustrating how the model captures stylistic lexical signals related to rhetorical language and descriptive imagery. The importance values indicate the contribution of each token to the model’s prediction.

In the *FrequentPoets* dataset, distinctive lexical patterns emerge that reflect the thematic and stylistic tendencies of individual poets. For Ahmad al-Safi al-Najafi, influential tokens include terms related to poetic language and rhetorical expression, such as “isti’ara” (metaphor), “lafz” (word or expression), and “wazn” (poetic meter). These tokens are closely connected to the discourse of poetic composition and literary criticism, suggesting that Najafi’s poetry frequently engages with metapoetic reflection on language, style, and artistic expression. The prominence of such tokens indicates that the model captures stylistic cues related not only to thematic content but also to explicit references to poetic structure and rhetorical technique.

A different lexical profile appears in the poetry of Khalil Mutran. Influential tokens extracted from Mutran’s verses include terms associated with geographical imagery and descriptive narration, such as references to the Nile and directional expressions such as the south. These lexical items reflect the descriptive and narrative character of Mutran’s poetic style, where landscape imagery and spatial references frequently appear as central motifs. The presence of such tokens suggests that the model captures stylistic signals related to vivid visual description and environmental imagery.

In addition to these lexical cues, several influential tokens correspond to subword units generated through the WordPiece tokenisation used in transformer architectures. This observation indicates that stylistic information may also be encoded through morphological patterns embedded within Arabic words. Given the rich morphological structure of Arabic, where stylistic nuance often emerges through derivational forms and affixes, such subword representations allow the model to capture subtle stylistic distinctions between poets.

**Fig. 7:**
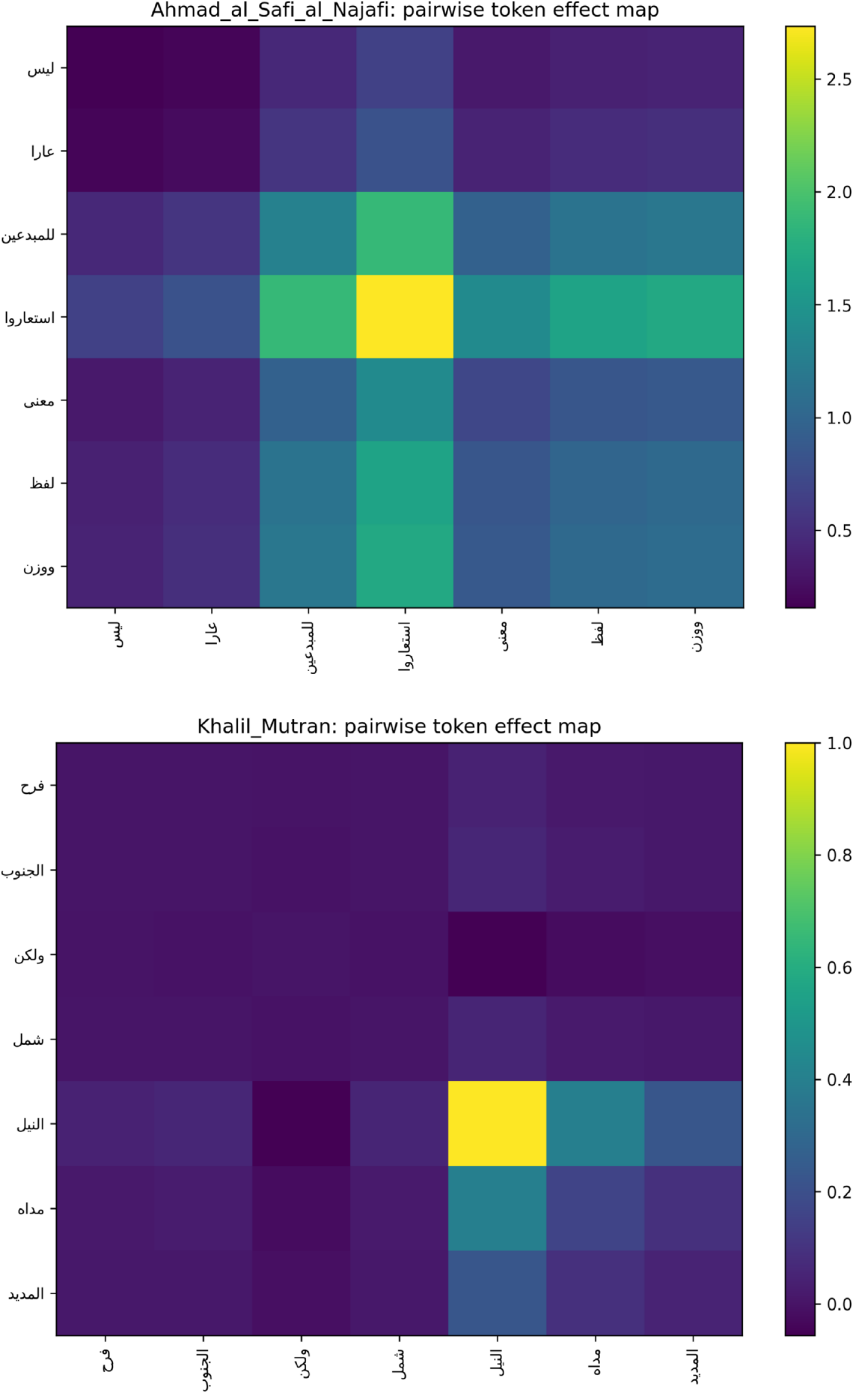
Pairwise token-effect heatmaps illustrating token interactions captured by AraPoemBERT. (a) Ahmad al-Safi al-Najafi. (b) Khalil Mutran. The colour intensity indicates the strength of interaction between influential tokens within the poet’s verses.

In addition, the token interaction heatmaps (Fig. 7) further illustrate how influential tokens co-occur within poetic contexts, providing complementary evidence to the token-level importance analysis. For example, in the case of Ahmad al-Safi al-Najafi, tokens related to rhetorical language such as “isti’ara”, “lafz”, and “wazn” exhibit strong interaction patterns, indicating that they frequently appear together and collectively contribute to the model’s prediction. Similarly, for Khalil Mutran, tokens associated with geographical imagery, such as references to the Nile and directional expressions, form coherent interaction clusters reflecting descriptive stylistic patterns. These interaction patterns indicate that the model captures not only individual lexical signals but also relationships between stylistic elements across tokens.

Taken together, these findings demonstrate that AraPoemBERT captures stylistic variation across multiple linguistic levels, including lexical choice, thematic preference, and morphological structure. Importantly, this analysis provides direct interpretability evidence that the model does not rely solely on superficial lexical frequency patterns, but instead learns meaningful stylistic representations that reflect both semantic content and rhetorical structure. This behaviour helps explain the model’s improved performance, as it can distinguish poets based on deeper stylistic patterns rather than isolated keywords.

### Representation analysis of hidden features

To further investigate how stylistic information is encoded internally, we analysed the hidden representations generated by AraPoemBERT and projected them into a two-dimensional space using Principal Component Analysis (PCA). The resulting projections are illustrated in Figs. 4 and 5.

The PCA projections reveal partially separable clusters corresponding to different poets, suggesting that the model organises verses within a continuous stylistic representation space in which stylistically similar poets tend to occupy neighbouring regions. In several areas of the projection, distinct author-specific groupings emerge, indicating that the model captures meaningful stylistic signals associated with particular poets.

However, the projections also reveal areas of partial overlap between clusters. These overlaps correspond closely to the misclassification patterns observed in the confusion matrices, suggesting that poets whose verses occupy nearby regions in the representation space are also more likely to be confused during classification. This correspondence suggests that the internal representation structure learned by AraPoemBERT may reflect underlying stylistic relationships between poets rather than arbitrary separations.

Interestingly, Ibn al-Rumi and Al-Ma’arri appear relatively close in the representation space. Although they belong to different historical periods, they both emerge from closely related phases of the classical Arabic literary tradition. This temporal and cultural proximity may partly explain stylistic similarities in vocabulary usage and rhetorical constructions captured by the learned representations of the model.

### Role of temporal diversity

Another important observation concerns the influence of temporal diversity in the dataset. Models consistently achieved higher accuracy on the *CrossEraPoets* dataset compared with the *FrequentPoets* dataset (see Tables 3 and 5). This pattern suggests that greater temporal diversity between poets may contribute to improved stylistic separability and facilitate more reliable classification.

When poets originate from clearly distinct historical contexts, differences in vocabulary, rhetorical style, and poetic conventions tend to become more pronounced. These distinctions provide clearer stylistic cues that allow the model to associate linguistic patterns with specific authors. Conversely, poets belonging to similar literary traditions may share broader stylistic conventions and overlapping lexical choices, which can make computational discrimination more challenging.

The observed performance differences therefore suggest that temporal variation in poetic language contributes to clearer stylistic boundaries in the representation space, ultimately supporting more accurate author classification.

### General-purpose LLM limitations

In contrast to the transformer models fine-tuned in this study, GPT-4o demonstrated substantially lower performance under both zero-shot and few-shot prompting settings in the present experiments. This result highlights the limitations of applying general-purpose LLMs directly to specialised literary tasks without domain-specific training.

Arabic poetry often contains complex morphological structures, dense figurative language, and historically grounded stylistic conventions^8^. that may not be strongly represented in the general training data of multilingual LLMs. Consequently, although GPT-4o exhibits strong general language understanding capabilities, it appears less able to capture the subtle stylistic cues required for reliable poet attribution.

### Impact of prompting strategies

The experiments also provide insight into how prompting strategies influence the behaviour of general-purpose large language models. When comparing zero-shot and few-shot prompting configurations for GPT-4o, a clear difference emerges between the two datasets. On the *FrequentPoets* dataset, the few-shot setup produced only a modest improvement over the zero-shot configuration. This limited gain suggests that providing a small number of labelled examples may not be sufficient when the poets share similar stylistic conventions and vocabulary.

By contrast, the improvement was substantially larger on the *CrossEraPoets* dataset. Because this dataset includes poets from different historical periods, stylistic distinctions between authors are more pronounced. In such cases, the labelled examples included in the prompt appear to provide stronger cues that help the model associate linguistic patterns with particular poets.

These findings highlight an important limitation of prompt-based inference for literary analysis tasks. Although few-shot prompting can partially improve performance, it does not replace the benefits of domain-specific training. Models that are explicitly pretrained or fine-tuned on poetic corpora remain better suited to capturing the subtle stylistic patterns required for reliable poet attribution.

### Limitations

Despite the promising outcomes reported in this study, several limitations should be acknowledged. First, the experiments were conducted on datasets containing a limited number of poets. Although the selected poets represent both prolific authors and cross-era stylistic variation, the datasets may not fully capture the broader diversity of Arabic poetic traditions.

Second, the linguistic scope of the datasets is primarily restricted to classical and Modern Standard Arabic poetry. Consequently, the findings may not directly generalise to other poetic forms such as dialectal poetry, contemporary free verse, or hybrid literary styles.

Third, GPT-4o was evaluated using prompt-based inference with both zero-shot and few-shot prompting strategies. The model was used in its out-of-the-box configuration without any task-specific training on poetic corpora.

Fourth, although the experiments demonstrate strong performance for domain-adapted transformer models, the results may still depend on the characteristics of the pretraining corpus, including its stylistic and historical distribution.

Finally, the experiments were conducted on specific datasets constructed for Arabic poet classification. Additional validation across independent poetic corpora would further strengthen the generalisability of the findings.

These limitations may affect the generalisability of the results, particularly when applying the models to more diverse poetic styles, dialectal variations, or unseen literary domains.

### Implications

Taken together, the findings demonstrate that domain-adapted transformer models provide a robust and effective computational framework for analysing stylistic variation in Arabic poetry. By combining comparative benchmarking with representation-level analysis, the study moves beyond a purely performance-oriented evaluation and offers insight into how stylistic and authorial signals may be encoded within modern language models.

These implications extend beyond the immediate task of poet classification. From an Arabic NLP perspective, the results highlight the importance of domain-specific pretraining when modelling linguistically rich, stylistically dense, and culturally specialised texts. More broadly, the study contributes to digital humanities research by supporting computational approaches to authorship attribution, stylistic interpretation, and the large-scale exploration of Arabic literary heritage.

The findings also suggest promising directions for future research. In particular, extending the analysis to larger and more diverse poetic corpora, incorporating contextual metadata such as historical era or poetic theme, and applying more detailed interpretability methods may further clarify how stylistic distinctions are represented within transformer-based models. Such developments could strengthen the role of computational methods not only in literary classification, but also in supporting deeper scholarly engagement with the evolution, structure, and diversity of Arabic poetic traditions.

## Ethical and cultural considerations

Arabic poetry is inseparable from cultural memory, identity, and spiritual expression. Automated poet classification should therefore complement—not replace—traditional literary scholarship. All sources in this study were limited to public-domain or openly licensed repositories; data handling adhered to fair-use norms and respected cultural provenance.

From an ethics-by-design perspective, model outputs must be contextualised to avoid reductive readings of poet intent, especially for devotional or regionally sensitive texts. We recommend releasing trained models with clear usage notes, documenting known failure modes (e.g., dialectal drift, genre shift), and providing opt-out mechanisms for contested attributions. Finally, collaboration with literary scholars and archivists is essential to ensure that computational pipelines support preservation, pedagogy, and equitable access to Arabic literary heritage.

All datasets and model implementation scripts developed in this study will be made publicly available on GitHub upon publication, under the MIT or CC BY-4.0 licence. All textual materials included in the datasets were verified to be in the public domain or distributed under open licences, ensuring full compliance with ethical and copyright standards.

## Conclusion

This study presented a comparative evaluation of transformer-based architectures, recurrent neural networks, and a general-purpose large language model for Arabic poet classification across two curated datasets: FrequentPoets and CrossEraPoets.

Across both datasets, transformer architectures consistently outperformed recurrent baselines, indicating the effectiveness of self-attention mechanisms for modelling the stylistic and contextual complexity of Arabic poetic language. Among all evaluated models, AraPoemBERT achieved the strongest overall results. On the FrequentPoets dataset, the model obtained approximately 73% test accuracy with stable validation performance. On the CrossEraPoets dataset, performance increased to approximately 77–78%, suggesting that greater temporal diversity between poets may improve stylistic separability and facilitate more reliable classification.

Importantly, the contribution of this study extends beyond simple performance comparison. Representation analysis based on PCA projections reveals that AraPoemBERT organises poetic verses within a structured stylistic feature space. Poet-specific regions emerge within this space, although partial overlap remains between stylistically similar authors. The degree of spatial overlap observed in the representation space corresponds closely to the misclassification patterns observed in the confusion matrices, indicating that stylistic similarity between poets is reflected directly in the geometry of the model’s hidden representations.

Token-level importance analysis further suggests that AraPoemBERT relies on linguistically meaningful cues, including both lexical items and morphologically informative subword units. This behaviour is particularly relevant for Arabic poetry, where stylistic signals often emerge through rich morphological constructions and recurring rhetorical patterns.

Overall, the findings suggest that domain-adapted transformer models provide an effective computational framework for analysing stylistic variation in Arabic poetry. By combining comparative benchmarking with representation-level analysis, this study contributes to the growing intersection between Arabic natural language processing and digital humanities. Future research may extend this work by incorporating additional poetic corpora, expanding the range of poets analysed, and developing deeper interpretability frameworks that connect internal model representations more directly to traditional literary concepts.

## Data Availability

The datasets generated and analysed during the current study are derived from publicly available Arabic poetry resources. The processed datasets used in this study (FrequentPoets and CrossEraPoets) and the source code for the experiments will be made publicly available upon acceptance of the manuscript. Until then, the data are available from the corresponding author upon reasonable request.

## Code availability

The code used in this study will be made publicly available upon acceptance of the manuscript at: https://github.com/scsaln/AraPoemBERT-Federated-Classification.
